# Long-Term Labeling of Hippocampal Neural Stem Cells by a Lentiviral Vector

**DOI:** 10.3389/fnmol.2018.00415

**Published:** 2018-11-15

**Authors:** Hoonkyo Suh, Qi-Gang Zhou, Irene Fernandez-Carasa, Gregory Dane Clemenson, Meritxell Pons-Espinal, Eun Jeoung Ro, Mercè Marti, Angel Raya, Fred H. Gage, Antonella Consiglio

**Affiliations:** ^1^Department of Neurosciences, Cleveland Clinic, Lerner Research Institute, Cleveland, OH, United States; ^2^Department of Pathology and Experimental Therapeutics, Institut d’Investigació Biomédica de Bellvitge, Bellvitge University Hospital, Barcelona, Spain; ^3^Institute of Biomedicine of the University of Barcelona, Barcelona, Spain; ^4^Gene Expression Laboratory, The Salk Institute for Biological Studies, La Jolla, CA, United States; ^5^Center of Regenerative Medicine in Barcelona, Hospital Duran i Reynals, Barcelona, Spain; ^6^Biomedical Research Networking Center in Bioengineering, Biomaterials and Nanomedicine, Madrid, Spain; ^7^Institució Catalana de Recerca i Estudis Avançats (ICREA), Barcelona, Spain; ^8^Department of Molecular and Translational Medicine, University of Brescia, Brescia, Italy

**Keywords:** lentiviral vectors, hippocampal neurogenesis, targeting, neural stem cells, lesion

## Abstract

Using a lentivirus-mediated labeling method, we investigated whether the adult hippocampus retains long-lasting, self-renewing neural stem cells (NSCs). We first showed that a single injection of a lentiviral vector expressing a green fluorescent protein (LV PGK-GFP) into the subgranular zone (SGZ) of the adult hippocampus enabled an efficient, robust, and long-term marking of self-renewing NSCs and their progeny. Interestingly, a subset of labeled cells showed the ability to proliferate multiple times and give rise to Sox2^+^ cells, clearly suggesting the ability of NSCs to self-renew for an extensive period of time (up to 6 months). In addition, using GFP^+^ cells isolated from the SGZ of mice that received a LV PGK-GFP injection 3 months earlier, we demonstrated that some GFP^+^ cells displayed the essential properties of NSCs, such as self-renewal and multipotency. Furthermore, we investigated the plasticity of NSCs in a perforant path transection, which has been shown to induce astrocyte formation in the molecular layer of the hippocampus. Our lentivirus (LV)-mediated labeling study revealed that hippocampal NSCs are not responsible for the burst of astrocyte formation, suggesting that signals released from the injured perforant path did not influence NSC fate determination. Therefore, our studies showed that a gene delivery system using LVs is a unique method to be used for understanding the complex nature of NSCs and may have translational impact in gene therapy by efficiently targeting NSCs.

## Introduction

Evidence from several studies has shown that self-renewing and multipotent neural stem cells (NSCs) are responsible for hippocampal neurogenesis, a process that maintains a NSC pool and generates newborn neurons (Gage, 2000; Suh et al., 2007; Eisch et al., 2008; Bonaguidi et al., 2011). This life-long production and integration of newborn neurons into the hippocampal neural circuits plays a crucial role in learning and memory as well as emotional and stress response (Deng et al., 2010). The regenerative capacity of NSCs has raised hopes that NSCs could be used to replace degenerating cells as a part of therapy for various neurodegenerative and neuropsychiatric disorders. This therapeutic potential of NSCs underscores the importance of understanding the properties of hippocampal NSCs.

The idea that hippocampal NSCs are multipotent and have a self-renewal capacity has been widely accepted. However, the details of these critical features of NSCs are still unclear. For example, although hippocampal NSCs can differentiate into both neurons and astrocytes, a series of fate-mapping studies and lineage analyses revealed that hippocampal NSCs produce predominantly neurons, whereas the generation of astrocytes and oligodendrocytes is minimal to non-existent (Seri et al., 2001; Kronenberg et al., 2003; Suh et al., 2007; Lugert et al., 2010; Bonaguidi et al., 2011; Encinas et al., 2011; Beckervordersandforth et al., 2014; Rolando et al., 2016; Pons-Espinal et al., 2017). However, it remains to be determined whether NSCs are continuously self-renewing over the long term or whether they are short-lived cells (Bonaguidi et al., 2011; Encinas et al., 2011; Beckervordersandforth et al., 2014). Both models can explain the presence of self-renewing NSCs, but they do not indicate whether NSCs are continuously self-renewing or live only for a short time. These inconclusive observations regarding key features of NSCs are partially due to the technical challenges involved in labeling and tracing complex NSC populations over time.

To address this issue directly, we used a lentivirus (LV)-mediated gene delivery system that has shown to transduce both dividing and non-dividing cells, including NSCs as well as their progeny over a long-term marking period (Consiglio et al., 2004; Suh et al., 2007). By taking advantage of LV’s ability to efficiently transduce a GFP reporter gene in adult NSCs *in vivo*, we clearly demonstrated the presence of long-lasting NSCs that can proliferate multiple times when spaced by month intervals and produce cells expressing a NSC marker, Sox2. Importantly, when we isolated, cultured, and examined the properties of GFP-labeled cells 3 months after we injected LV PGK-GFP into the dentate gyrus (DG), we observed that the same population labeled by LV PGK-GFP *in vivo* could expand and produce neurons as well as astrocytes *in vitro*. Using a LV PGK-GFP, we subsequently tested whether hippocampal NSCs underlies the burst of astrocyte formation that occurs during performant path (PP) injury. In the PP injury model that induces astrocyte production in the hippocampus (Gage et al., 1988; Fagan and Gage, 1994), we found that NSCs did not contribute to the burst of astrocytes. Our studies demonstrate that a LV is a robust and efficient gene delivery system that allows us to dissect the detailed characteristics of hippocampal NSCs.

## Materials and Methods

### LV Production and Stereotactic Surgery

The production and determination of LV PGK-GFP expression were previously described in detail (Consiglio et al., 2004). We used LV PGK-GFP that has 5 × 10^9^ to 1 × 10^10^ transducing units per ml with an HIV-1 p24 concentration of 100 μg/ml. All procedures using animals were done in accordance with protocols approved by the Animal Care and Use Committee of the Salk Institute for Biological Studies and the Parc Cientific of Barcelona. Six- to eight-week old female C57BL/6 (Harlan) mice were used for all experiments. Mice were anesthetized with a mixture of ketamine (100 mg/kg) and xylazine (10 mg/kg), and 1 μl of vector was injected into the right hippocampal dentate gyrus at a rate of 0.2 μl/min using a Hamilton (Reno, NV, United States) syringe. Stereotactic coordinates were as follows: AP = -2, ML = +1.5, DV = -2 in mm from bregma. Mice were analyzed 15 days and 3 and 6 months after injection.

### BrdU Administration

To understand the long-term proliferation of GFP-transduced cells in the adult hippocampus, 100 mg/kg of Bromodeoxyuridine (BrdU) was administrated daily by intraperitoneal (IP) injection for 7 days [83 and 173 days post injection (dpi)]. Animals were sacrificed 24 h after the last BrdU injection (90 and 180 dpi). To examine the differentiation potential of GFP-labeled cells, a group of mice received the same amount of BrdU at 30 dpi for 7 days and were analyzed 4 weeks later.

### Tissue Processing and Immunohistochemistry

Detailed procedures regarding tissue processing and antibody staining, including BrdU detection, were previously described (Suh et al., 2007). Primary antibodies used were mouse anti-Neuronal Nuclei (NeuN 1:10; kindly provided by Dr. R. Mullen, University of Utah); mouse anti-Nestin (1:500; Pharmingen); goat anti-Doublecortin (DCX 1:200; Santa Cruz Biotechnologies); rabbit anti-Glial Fibrillary Acidic Protein (GFAP 1:1000; Dako); rabbit anti-S-100β (1:5000; Swant); rat anti-BrdU (1:200; Accurate Chemicals); rabbit anti-Ki67 (1:200; Novocastra); rabbit anti-Sox2 (1:200, Chemicon); rabbit anti-brain lipid binding protein (BLBP 1:1000; kindly provided by N. Heintz, Rockefeller); rat anti-MUSASHI-1 (1:1000; a kind gift from O. Hideyuki, Keiyo University, Japan); rabbit anti-GFP (1:100, Molecular Probes); and guinea pig anti-GFAP (1:1,000). Fluorescence immunohistochemistry (IHC) was performed using corresponding FITC, Cy3, or Cy5 secondary antibodies (1:200, all raised in donkey, Jackson ImmunoResearch, West Grove, PA, United States). DAPI (10 mg/ml, Sigma) was used as a fluorescent counterstain.

Confocal stack images of brain slices (40 μm) were obtained with the Confocal A1 Nikon Inverted SFC with 40× objective and the Zeiss Spinning Disk with a 20× objective. Cell quantification and analysis was performed using NIS-Elements software (Nikon) and Zen Blue (Zeiss).

### Cell Counting

To quantify the percentage of labeled cells in coronal sections stained for GFP and BrdU, the number of BrdU-positive nuclei was identified in a selected optical field and counted. The fraction of labeled nuclei showing GFP-positive cytoplasm in the same field was then assessed. The analysis was performed on three randomly chosen fields taken from two or three independent non-sequential sections from three mice per experimental group.

### Perforant Path (PP) Lesion

Lentiviral vector expressing a green fluorescent protein was injected into the right side of the dentate gyrus of six- to eight-week old C57BL6 female mice (*n* = 5). One month later, these mice received ipsilateral aspirative lesions that resulted in the ablation of the PP as previously described (Fagan and Gage, 1994). Brains were harvested and analyzed 7 days post-surgery.

### Preparation of Hippocampal Progenitors

Six- to eight-week old C57BL/6 female mice were injected with LV PGK-GFP in the DG. Three months later, hippocampi were isolated from these mice and used to prepare hippocampal progenitor cells as previously described (Ray and Gage, 2006; Suh et al., 2007). Briefly, 3–5 LV PGK-GFP injected hippocampi were isolated from these mice and digested in PPD solution [papain (2.5 U/ml, Worthington), pronage (1 U/ml, Roche), and DNase (250 U/ml, Worthington)] and sucrose gradient was applied to remove cell debris and myelin. Then cells were plated in the presence of FGF2 (Fibroblast Growth Factor, 20 ng/ml; PeproTech), EGF (Epidermal Growth Factor, 20 ng/ml; PeproTech), and heparin (5 μg/ml; Sigma) in DMEM/F12 (Life Technology) basal media supplemented with N2 (Life Technology). Once NSCs were established and expanded in the presence of FGF2 and EGF, we sorted out GFP-expressing cells via FACS and established three clonally expanded NSCs lines. The number of total GFP-expressing cells isolated by FACS was around 2 × 10ˆ4 cells per preparation. To differentiate GFP-expressing NSCs, 10^5^ cells/cm^2^ were plated on the laminin-coated glass chamber slides (Nalge Nunc International) and cultured in differentiation medium consisting of DMEM/F12, N2 supplement, and 5 μM forskolin (Sigma), for 7 more days.

### Immunofluorescence

The immunofluorescence staining on cell cultures was performed after fixing NSCs for 30 min with 4% paraformaldehyde (PFA) followed by extensive washings with PBS for 30 min. Cells were washed three times with PBS 0,1% Triton X-100 (PBS-T) and blocked for 2 h with PBS-T containing 5% normal goat serum (Vector laboratories), followed by overnight incubation with primary antibodies: rabbit anti- GFAP (1:1000; Dako); mouse anti-Nestin (1:500; Pharmingen); rabbit anti-Sox2 (1:200; Chemicon); anti-TUJ-1 (1:1,000; Chemicon). The next day, after washing extensively with PBS-T, cells were incubated with secondary antibodies. Cells were mounted in mounting medium and counterstained with fluorescent nuclear dye DAPI (Invitrogen). Images were obtained using the microscope Nikon Eclipse at 20 or 40× magnification and quantification was performed using a Cell-counter plugin in FIJI (Fiji is Just ImageJ).

## Results

### LV-Mediated Long-Term Labeling of NSCs in the Hippocampus

**FIGURE 1:**
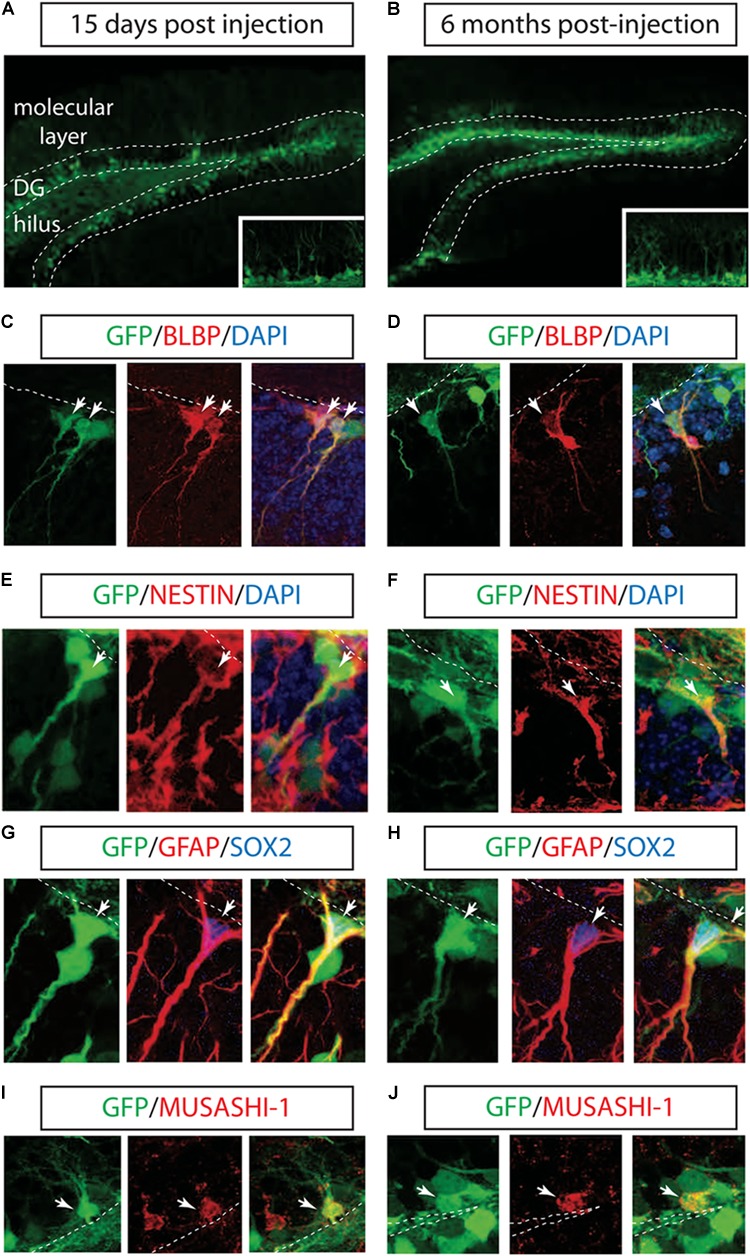
Long-term marking of hippocampal NSCs by LV PGK-GFP. LV PGK-GFP was unilaterally injected into the hippocampal DG; brain sections were analyzed 15 days **(A)** and 6 months **(B)** later. GFP expression was evident in the DG at both time points. A higher magnification view is displayed in insets **(A,B)**. GFP-expressing cells co-labeled with NSCs markers such as BLBP **(C,D)**, NESTIN **(E,F)**, SOX2, GFAP **(G,H)**, and MUSASHI-1 **(I,J)** (arrows). Note that some GFP-positive cells stained for SOX2 showed co-localization with radial glial cell markers such as GFAP in their processes **(G,H)**. DG, dentate gyrus; SGZ is marked with dotted lines.

To test the ability of LV to transduce hippocampal NSCs *in vivo*, we injected a vesicular stomatitis virus-pseudotyped, late-generation LV expressing the GFP into the right DG of the mouse hippocampus (Consiglio et al., 2004). GFP expression in the LVs was driven by the ubiquitously expressed phosphoglycerate kinase promoter and by the posttranscriptional regulatory element of the woodchuck hepadnavirus. Mice were sacrificed after 2 weeks to assess cell phenotype shortly after transduction or after 3 and 6 months as the longest time point. Robust GFP-expressing cells were identified in both DG and the hilus after 2 weeks of injection and during a six-month period (Figures 1A,B). Because neither the VSV-G envelope nor the PGK promoter provided neural precursor cell (NPCs) specificity, the majority of GFP-labeled cells expressed post-mitotic immature and mature neuronal markers (data not shown).

Next, we tested whether the LV successfully labeled hippocampal NSCs. Interestingly, IHC with NSCs markers showed that a population of GFP^+^ cells located along the subgranular zone (SGZ) co-localized with BLBP, NESTIN, SOX2, GFAP, and MUSASH-1 2 weeks and 6 months after LV PGK-GFP injection (Suh et al., 2007; Figures 1C–J).

Three-dimensional confocal images revealed that 38 ± 5.3% (*n* = 3) of the GFP-positive cells in the SGZ expressed BLBP marker, known to label a subset of neurogenic radial glia (Filippov et al., 2003; Fukuda et al., 2003; Mignone et al., 2004; Gebara et al., 2016), and 7 ± 2.2% (*n* = 3) was expressed in non-radial cells (SOX2^+^ cells), suggesting the ability of LV PGK-GFP to target NSCs that have the potential to self-renew and differentiate into neurons in the adult hippocampus (Suh et al., 2007; Bonaguidi et al., 2011; Gebara et al., 2016).

### LV Marks a Cell Population That Retains Multiple Proliferation Capacity

**FIGURE 2:**
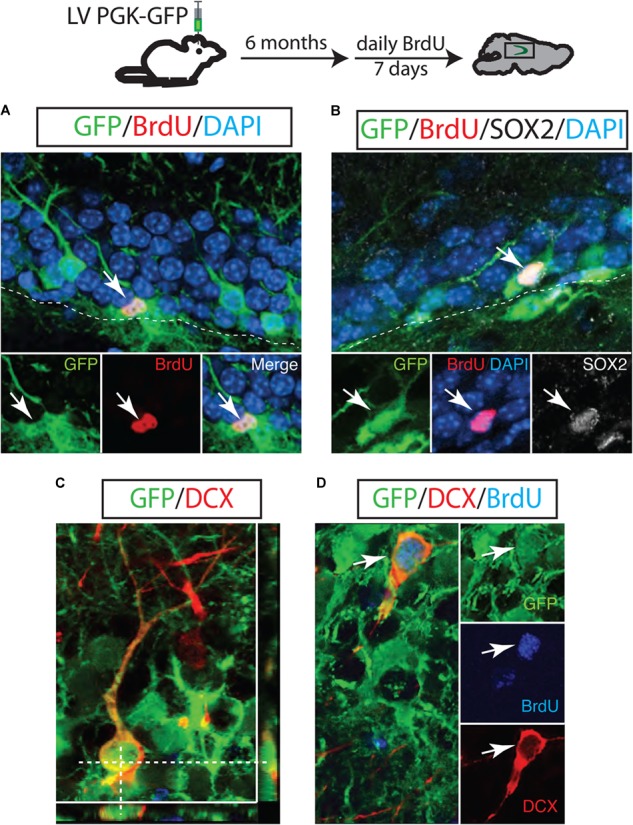
A long-lasting NSC population in the adult hippocampus. GFP-positive neuroblasts were observed in the SGZ 6 months after LV PGK-GFP injection. Some GFP^+^ cells incorporated BrdU **(A)** and maintained the expression of SOX2 **(B)**, indicating that LV PGK-GFP-labeled NSC populations retained the proliferation capacity over a six-month tracing period. Many of the GFP-labeled cells expressed the early neuronal marker doublecortin (DCX; **C**). Higher magnification picture of the triple-labeled cells GFP/DCX/BrdU **(D)**.

To test whether LV PGK-GFP is successfully transduced in NSCs that have proliferation capacity over a long period of time, we introduced BrdU 6 months post LV PGK-GFP injection. The hippocampus was then examined to identify proliferating cells 24 h after the final BrdU injection. We found that 6.25 ± 1.8% of GFP^+^ cells were positive for BrdU in the SGZ of the DG. This observation indicates that initially targeted cells or their progeny retain proliferation capacity over 6 months (Figure 2A). We identified that 10 ± 1.4% of these GFP/BrdU double-labeled cells expressed SOX2 marker (*n* = 3; Figure 2B), known to represent the self-renewing and multipotent NSCs (Suh et al., 2007). Doublecortin (DCX), a transient marker that labels both neuroblasts and immature neurons, was used to measure a continuous neurogenesis from GFP^+^ cells (Steiner et al., 2006). Indeed, we observed both GFP/DCX double-labeled cells (26.8 ± 2% of GFP^+^ cells; *n* = 3 Figure 2C) and GFP/BrdU/DCX cells triple-labeled cells (60 ± 2.3% *n* = 3; Figure 2D). Interestingly, these results collectively suggest that NSCs are continuously generated from targeted GFP^+^ cells and actively produce neuroblasts even 6 months after LV injection.

### Long-Term Maintenance of NSCs in the Hippocampus

**FIGURE 3:**
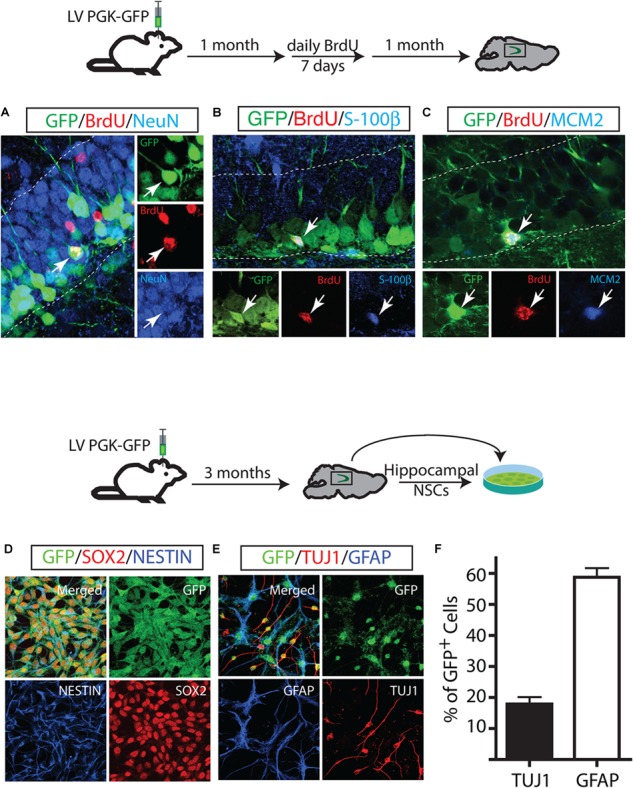
Long-term maintenance of NSCs in the adult hippocampus. Fate mapping of GFP^+^ identified NSCs that proliferate and produce neurons **(A)** and astrocytes **(B)**. Some NSCs underwent cell proliferation proliferated twice in a one-month interval **(C)**. GFP-labeled cells *in vivo* gave rise to *in vitro* NSCs. *In vitro*, GFP^+^ NSCs expressed NSC markers such as NESTIN and Sox2 **(D)** and differentiated into neurons (TUJ1) and astrocytes (GFAP) **(E)**. GFP^+^ derived-neurons and astrocytes at day 7 of differentiation **(F)**.

Next, we investigated the cell fate of the proliferating GFP^+^ cells and their extensive proliferation ability. One month after the LV PGK-GFP injection, mice were injected with BrdU daily for 7 days and sacrificed 4 weeks after the last BrdU injection. The majority of progeny of proliferating GFP^+^ cells (GFP/BrdU double-labeled cells) gave rise to 87.5 ± 2.5% neurons which account for 40% DCX^+^ and 60% NeuN^+^ (Figure 3A). Specifically, neurons positive for NeuN expressed a granular neuron-specific marker, Prox1 (data not shown) and displayed distinctive morphological features of mature granular neurons in the DG. In contrast to a dominant neuronal differentiation of GFP-labeled cells, proliferating GFP^+^ (GFP/BrdU^+^) cells produced a significantly lower number of mature astrocytes that co-labeled with S-100β (3.9 ± 0.49% GFP/BrdU/S-100 β+ cells) (Figure 3B); the generation of new oligodendrocytes was not detected in our experimental paradigm. These results are in line with those obtained by using other labeling methods (Song et al., 2002; Maslov et al., 2004; Steiner et al., 2004; Suh et al., 2007, 2009; Zhao et al., 2008; Venere et al., 2012). We also performed IHC with BrdU and MCM2 to test the proliferation capacity of GFP^+^ cells (Maslov et al., 2004). A triple staining revealed a subset of GFP/BrdU double positive cells expressing MCM2 (2.3 ± 1.2%), indicating that GFP^+^ cells had proliferated 1 month after LV injection and were in continuous cell cycle for an additional month (Figure 3C).

Our assessment of the differentiation capacity of GFP^+^ cells *in vivo* was in sharp contrast to the multipotency capacity of the hippocampal NSCs *in vitro* that has been reported in many studies: while GFP^+^ cells *in vivo* differentiated predominantly to neurons, cultured NSCs produced more astrocytes than neurons. This finding raised the possibility that neurogenic cells labeled by LV PGK-GFP might differ from the cell population that gives rise to hippocampal NSCs *in vitro*. To test this hypothesis, we isolated the hippocampal NSCs 3 months after we injected LV PGK-GFP into the DG and established NSCs with clonally derived GFP^+^ cells. Clonally expanded GFP^+^ NSCs showed their ability to propagate at least 20 times, maintaining the expression of NSC markers of NESTIN and SOX2 (Figure 3D). When the NSCs were induced to differentiate in the presence of forskolin, the majority of GFP^+^ NSCs differentiated into astrocytes positive for GFAP, although a moderate amount of GFP+ NSCs gave rise to neurons positive for Tuj1 (Figures 3E,F). These results showed that LV PGK-GFP clearly targeted the same cell populations that serve as a source of both *in vitro* and *in vivo* NSCs, though the *in vitro* conditions favored astroglial differentiation.

### Differentiation Potential of NSCs in the Perforant Path Injury

It has been shown that unilateral injury of the perforant path (PP) induces degeneration of axon terminals of the entorhinal neurons and a burst of astrocyte formation in the molecular layer of the ipsilateral hippocampus (Gage et al., 1988; Fagan and Gage, 1994). It has been a long lasting question whether NSCs in the dentate gyrus could contribute to the PP injury-induced astrocytes. To directly answer to this question, 1 month after LV PGK-GFP was injected to label NSCs in the DG, a unilateral transection of the PP was performed. BrdU was injected for 3 days and the brain was analyzed 4 days later.

**FIGURE 4:**
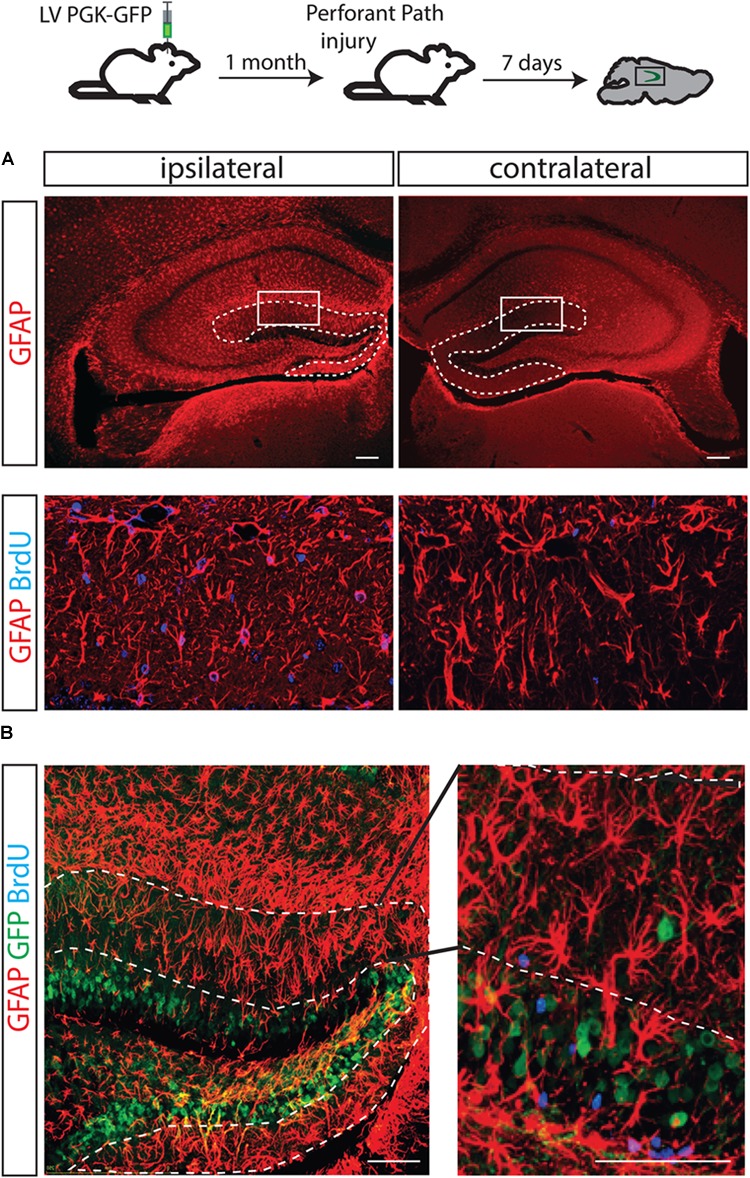
The fate of hippocampal NSCs did not change following PP injury. A brain lesion in the perforant path resulted in over-expression of GFAP-positive cells in the molecular layer of the injury side but not the contralateral side (**A**, dotted line). As expected, an increased number of astrocytes (positively stained with BrdU) was found in the molecular layer of the ipsilateral side (**B**, left) but not in the contralateral side of these animals (**B**, right); however, GFP-labeled progenitors did not contribute to the increased astrocyte numbers.

Consistent with previous reports, the generation of astrocytes expressing GFAP increased on the ipsilateral side of the molecular layer (Figure 4A). Thus, by immunofluorescence analyses, we confirmed that the ablation of inputs from entorhinal cortex to the granular neurons causes degeneration of axons and results in increased astrocyte proliferation (GFAP/BrdU double positive cells), specifically in the DG-molecular layer of the lesioned side (Figure 4A). However, the contribution of GFP^+^ cells to newly generated astrocytes was not detected, suggesting that GFP^+^ NSCs were not responsible for the burst of astrocytes (Figure 4B).

## Discussion

Self-renewing and multipotent NSCs are responsible for continuous neurogenesis in the adult hippocampus. These two properties of NSCs are critical for the production of newborn neurons and maintenance of a stem cell pool throughout life. As neurogenesis occurs, adult NSCs undergo changes in their intrinsic properties such as morphology, gene expression, proliferation kinetics, and self-renewal capacity and ultimately produce differentiated neural cells (Zhao et al., 2008; Suh et al., 2009). In the normal brain, the production, and integration of newborn neurons into the preexisting neural circuits play a key role in hippocampus-dependent learning and memory (Aimone et al., 2011). In pathological conditions, however, injury-associated signals appear to affect many aspects of neurogenesis, leading to changes in the proliferation of NSCs, survival and fate determination of newborn cells (Lindvall and Kokaia, 2006; Llorens-Bobadilla et al., 2015). Dysregulated neurogenesis has been implicated in functional deficits. In this report, we investigated the precise characteristics of LV-targeted NSCs to understand how NSCs contribute to neurogenesis in normal and injured brains.

Two different models have been proposed to address the nature of the longevity of NSCs in the adult hippocampus: long-lasting and continuously self-renewing NSCs vs. one-time activated and short-living NSCs. In the former model, NSCs continuously proliferate to maintain a NSC pool and produce newborn neurons and astrocytes (Suh et al., 2007; Bonaguidi et al., 2011). In the latter model, however, NSCs are proposed to be activated only once throughout life, to proliferate a limited number of times and sequentially generate neurons and astrocytes within a period of a month (Encinas et al., 2011). This latter model of short-lived NSCs is in line with previous *in vitro* studies, suggesting that the adult hippocampus does not contain NSCs but only lineage-committed and transiently proliferating cells (Seaberg and van der Kooy, 2002; Bull and Bartlett, 2005). The key difference between these models is whether NSCs have a long-term or short-term self-renewal ability. Here, using an advanced-generation LV (Consiglio et al., 2004), we showed that a subpopulation of GFP^+^ cells (indicating lentivirus-mediated labeling), retained the proliferative capacity over a six-month labeling period analyzed. Moreover, some GFP^+^ cells divided multiple times (GFP/BrdU/MCM2) within a one-month interval, producing progeny expressing a NSC marker (GFP/BrdU/SOX2) over the two-month period analyzed. That NSCs were targeted *in vivo* by LVs was further validated by culturing experiments demonstrating that some of the transduced SGZ cells behaved as long-term self-renewing progenitors *in vitro* and they could propagate for up to 5 months (the latest time tested). Interestingly, when we analyzed the differentiation ability of the labeled cells *in vitro*, our results showed that while GFP^+^ cells *in vivo* differentiated predominantly to neurons, cultured NSCs produced more astrocytes than neurons, indicating that *in vitro* conditions favored astroglial differentiation. When we clonally expanded GFP+ NSCs that had already been established *in vitro*, they all showed the similar differentiation ratio of neuron and astrocytes, suggesting this preferred astrocyte differentiation is likely to reflect the general NSC properties *in vitro*.

Using our LV system, we directly asked whether NSCs in the DG directly contributed to astrocytes formation by performing a unilateral transection of the PP, which induces astrocyte production specifically in the molecular layer of the ipsilateral DG (Gage et al., 1988; Fagan and Gage, 1994). Since the site of astrocyte induction is geographically separated from the injury site, our experimental paradigm has the advantage of ruling out the possibility that the cellular phenotypes we observed were caused by the direct physical damage to the brain. In such an experimental condition that facilitates astrocyte induction, we predicted we would observe increased astrocyte formation derived from NSCs labeled by a lentiviral vector. Contrary to what was expected, our fate mapping showed that NSCs did not contribute to the formation of astrocytes. LV-mediated fate mapping revealed that the fate determination of NSCs might be less influenced by environmental cues in the injured brains, and astrocyte progenitors located in the molecular layer might be responsible for astrocyte induction in the case of the PP injury model (Buffo et al., 2008; Zhang and Barres, 2010).

Although these findings support the conclusion that our lentiviral system is able to induce transgene expression in a long-lasting, self-renewing NSCs within the hippocampus of the adult mouse brain that continually give rise to proliferating neuronal progenitors that contribute to maintain neurogenesis in the hippocampus, the fraction of NSC that were actually transduced *in vivo* remains to be determined. An unequivocal characterization of the cell type originally transduced by the LV may require the use of cell type specific promoters restricted to NPCs. However, in spite of its uncertain identity, our findings indicate that LV, in combination with BrdU-labeling and morphological analysis, allows long-term studies of adult NSCs properties *in vivo*. In future experiments, we will test whether the combination of the usage of cell type specific promoters and different glycoprotein will improve targeting specificity, which is a critical factor also for successful gene therapy.

## Author Contributions

HS, FG, and AC conceived the study. HS, Q-GZ, GC, IF-C, and MP-E contributed to methodology. HS and AC performed the formal analysis. HS, Q-GZ, GC, IF-C, MP-E, ER, AR, MM, and AC investigated the analysis. HS, FG, and AC validated the results. HS, FG, and AC wrote, reviewed, and edited the manuscript. HS, FG, and AC acquired funding. HS, FG, and AC supervised the study. All authors edited and approved the final manuscript.

## Conflict of Interest Statement

The authors declare that the research was conducted in the absence of any commercial or financial relationships that could be construed as a potential conflict of interest.
